# Molecular engineering of individual dye-based nanoparticle photostability for ultrabright two-photon fluorescence

**DOI:** 10.3762/bjnano.17.48

**Published:** 2026-05-22

**Authors:** Eleonore Kurek, Sasha Cooper, Alexandre Clausolles, Karen Perronet, Jonathan Daniel, Mireille Blanchard-Desce, François Marquier

**Affiliations:** 1 Univ. Bordeaux, CNRS, Bordeaux INP, ISM (UMR5255), 351 Cours de la Libération, 33405 Talence, France; 2 Université Paris-Saclay, École Normale Supérieure Paris-Saclay, CNRS, CentraleSupélec, LuMIn, 91190 Gif-sur-Yvette, France

**Keywords:** bottom-up design, organic nanoparticles, two-photon fluorescence, two-photon microscopy

## Abstract

Dye-based fluorescent organic nanoparticles (dFONs) represent a promising class of bioimaging probes combining high brightness with molecular tunability. While their fluorescence performance is well established for one-photon excitation, their single-particle properties under two-photon excitation remain to be evaluated. Here, we perform a comprehensive optical characterization of two distinct dFONs designed to exhibit the same two-photon brightness but with different photostabilities. Saturation measurements of individual nanoparticles allowed for an estimation of their two-photon absorption cross sections, which were found to be consistent with ensemble values when taking into account a local-field correction. Time-resolved experiments further revealed that nanoparticles with the highest absorption cross section photobleach significantly faster, confirming the trade-off between absorption efficiency and photostability. These results demonstrate that the photophysical behavior of dFONs can be rationally engineered at the molecular level and provide design principles for the development of optimized organic nanoparticles for nonlinear fluorescence microscopy and bioimaging applications.

## Introduction

Dye-based fluorescent organic nanoparticles (dFONs) are a class of self-stabilized bioimaging probes composed solely of aggregated hydrophobic dye molecules. Their most notable asset is their exceptional brightness, which is the product of their one- or two-photon absorption cross sections, their fluorescence quantum yield, and the number of dye molecules per nanoparticle [1]. Thanks to the ultimate confinement of a large number of dye molecules and to molecular engineering strategies to maintain fluorescence in the aggregated state, such dFONs can indeed show giant brightness (typically up to 10^8^ M^−1^·cm^−1^, for nanoparticles measuring 30 to 40 nm in diameter) [2–3]. Even more importantly, the optical and chemicophysical properties of dFONs (including their colloidal stability and surface properties) can be precisely tuned through molecular engineering of specific dye building blocks [1]. dFONs have demonstrated significant potential in bioimaging applications. They have been used as biosensors for various ions [4] and thiols [5], as bioactuators for drug delivery [6–9], and most recently as actuators for optogenetics in mice [10]. However, dFONs are most widely used as biomarkers for fluorescence imaging. Their stealth properties can be engineered as needed [11–14], making them versatile for use in cells, tissues [15] and whole animals, such as *Xenopus* tadpoles [3], zebrafish [16–17], and mice [15]. Furthermore, dFONs have proven highly effective as probes for single-particle tracking (SPT) experiments, both within cells [18–19] and deep in the extracellular space of the mouse brain [20]. Recently, we achieved a milestone by performing 3D real-time two-photon-excited SPT on dFONs [21], using the 3D-Redshot setup [22]. In this case, dFONs proved particularly effective compared to more commonly used inorganic probes because they combine a large two-photon (2P) brightness with a small size, allowing for unprecedented localization precision of around 3 nm. In this article, we will show in particular that the dFONs brightness is larger than the sum of the brightnesses of the individual dyes. We experimentally demonstrate that 2P absorption from an individual dye is much more efficient in the nanoparticle than in solution. This effect can be explained by a local-field correction factor in the nanoparticle.

However, one of the main challenges of using organic probes such as dFONs in SPT experiments, compared to inorganic probes like gold nanoparticles [23], fluorescent nanodiamonds [24–25], quantum dots [26], or inorganic nanocrystals [22], remains their limited photostability. Repeated excitation inevitably leads organic molecules to undergo photobleaching, an irreversible process through which they lose their ability to fluoresce. This can occur through various mechanisms, including the cleavage of covalent bonds following a transition to the triplet state, and can be further accelerated by the presence of oxygen and the generation of reactive oxygen species (ROS) [27]. The resulting loss of fluorescence can significantly shorten tracking durations while compromising the localization precision, as the signal from the nanoparticles decreases over time [21]. Strategies to limit photobleaching may include reducing the excitation intensity, or limiting oxygen reaction, for example by removing oxygen through degassing or by adding oxygen scavengers [27]. However, these approaches can compromise the quality of the collected data or disrupt the biological environment under study. A more promising alternative is to seek to limit photobleaching through molecular engineering. Indeed, the photobleaching of organic molecules has been found to vary widely depending on their structure [28].

In the case of dFONs, a common practice is to assess the overall photostability of the nanoparticles in solution [16–17,29–30] or in cells [15,29], by monitoring the fluorescence intensity under continuous illumination. These studies effectively demonstrate the superior photostability of dFONs compared to individual organic dye molecules. This improvement arises both from the large number of dye molecules incorporated within the nanoparticles [19] and from the protection offered to the interior dye against water, as similarly observed in other dye-doped nanoscale systems [31]. However, such ensemble measurements fail to reveal the photobleaching behavior of individual dFONs. This was pioneered by our team, by tracking the fluorescence intensity of single dFONs over time during one-photon-excited wide-field video-microscopy SPT experiments. The resulting exponential decay plots revealed characteristic photobleaching times ranging roughly from 10 to 100 s, depending on the nature of the dFONs and their molecular building blocks [18–19].

Exploring the photostability of dFONs is especially relevant for 2P-excited SPT techniques, which require very high laser intensities, significantly increasing photobleaching by increasing the excitation rate. While several studies have investigated the 2P-excited photobleaching of organic dyes, to the best of our knowledge, no statistical analysis has addressed the possibility to engineer the photobleaching of individual dFONs under 2P excitation. In this paper, we selected two families of dFONs, previously reported in the literature [11,20]. They display the same 2P brightness, but differ notably in the fluorescence quantum yields and 2P absorption cross sections of their constituting dyes. By comparing the two systems, we experimentally reveal how these two parameters influence 2P-excited photobleaching at the individual nanoparticle level – an essential step toward the rational design of photostable dFONs.

## Results and Discussion

### Nanoparticles structure

**Figure 1:**
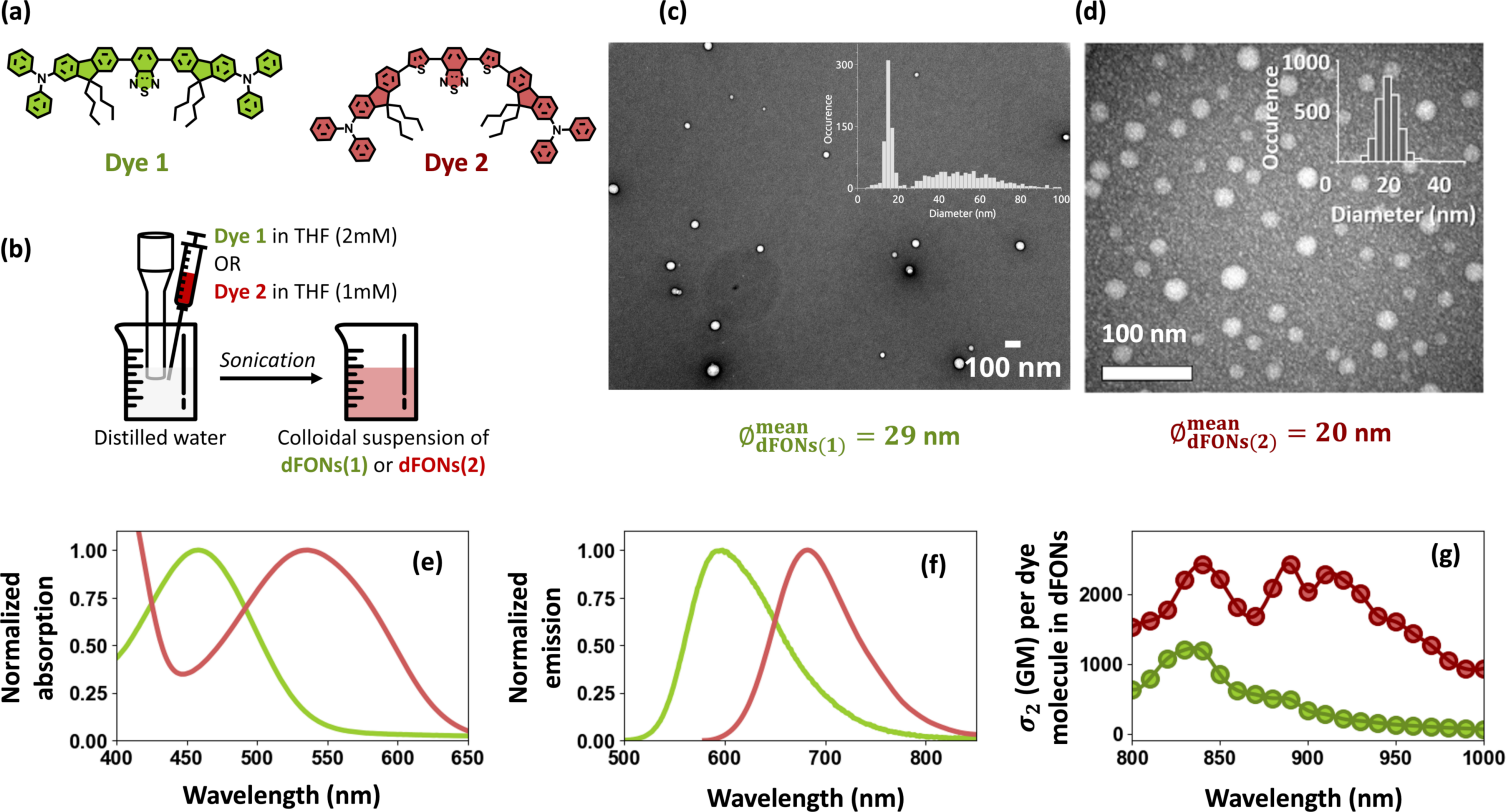
(a) Molecular structures of dyes 1 and 2. (b) Preparation scheme of dFONs(1) and dFONs(2) by precipitation, using, respectively, 2 mM and 1 mM stock solutions of dye 1 and dye 2. (c) Transmission electron microscopy (TEM) and size distribution of dFONs(1), data reused from [20]. (d) TEM and size distribution of dFONs(2). (e) Normalized absorption spectra of dFONs(1) (green) and dFONs(2) (red). (f) Normalized emission spectra of dFONs(1) (green) and dFONs(2) (red). (g) Two-photon absorption cross sections for one dye 1 (green) or dye 2 (red) molecule, measured as dFONs molecular subunits of respectively dFONs(1) and dFONs(2) [11]. The beaker image in Figure 1b was reproduced from “Beaker” icon, Copyright 2019 by CHARIE Tristan from thenounproject.com, distributed under the terms of the Creative Commons Attribution 3.0 Unported License (https://creativecommons.org/licenses/by/3.0/). Figure 1d was adapted with permission from [11]. Copyright 2021 American Chemical Society. This content is not subject to CC BY 4.0

**Figure 2:**
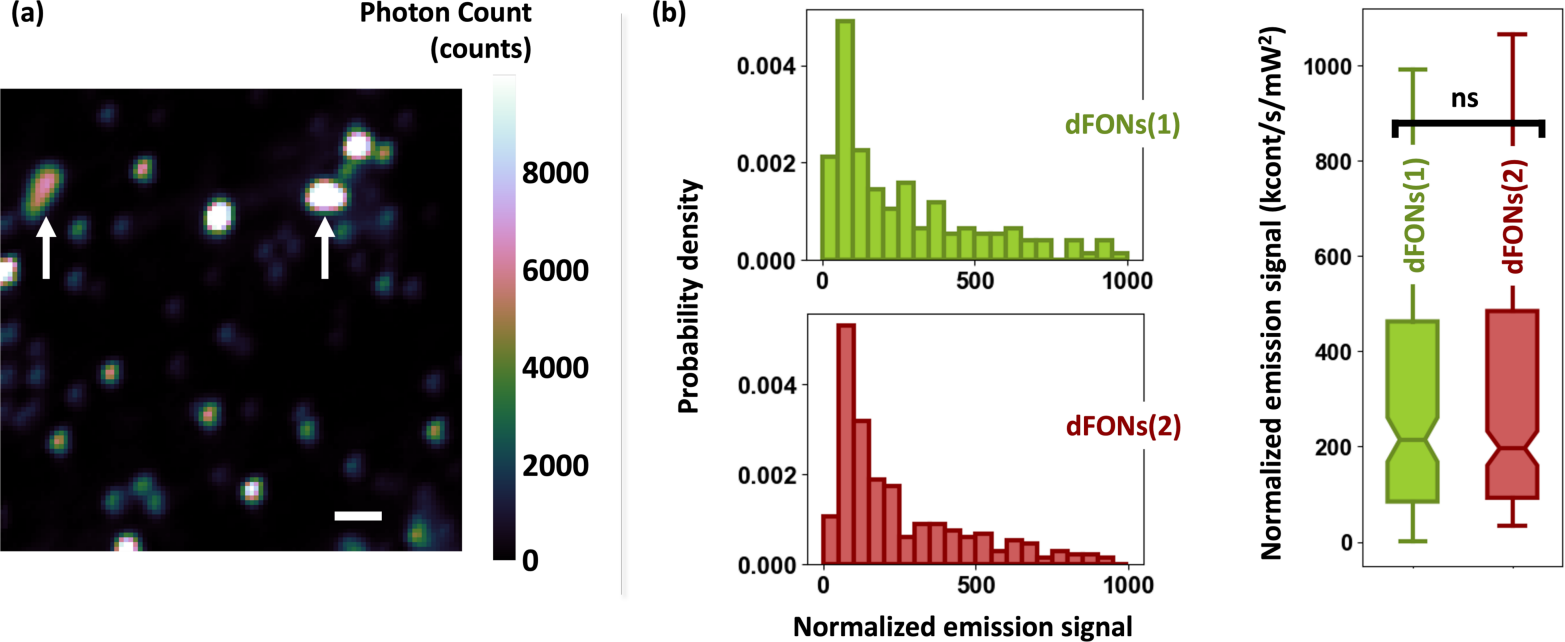
(a) Example of a scan (dFONs(1)) over a 10 μm × 10 μm region of interest (scale bar: 1 μm, pixel size: 100 nm × 100 nm, integration time per pixel: 10 ms). The brightest spots are saturated to reveal the nanoparticles with the lowest emission levels. The two arrows indicate two possible aggregates in the scan (shape and/or size different from the PSF). Each dFON leads to a PSF that can be fitted by a 2D Gaussian function. Its maximum defines the emission signals reported in panel (b). The signal levels are normalized by the square of the excitation power, and the units are kcounts·s^−1^·mW^−2^. (b) Histograms of emission signals from 293 dFONs(1) nanoparticles (green) and 193 individual dFONs(2) nanoparticles (dark red), and comparison of the distributions as a boxplot. The differences between the two distributions are statistically non-significant (*p*-value of 0.90).

Two families of dFONs, hereafter called dFONs(1) [20] and dFONs(2) [11], are obtained through precipitation [32] from quadrupolar dyes, hereafter called 1 and 2 (Figure 1a,b). By adjusting the dye concentration during precipitation [20], we tuned the nanoparticle diameters as can be seen in Figure 1c,d. Whereas dFON(2) exhibits a single size population centered at 20 nm, dFON(1) shows two distinct size populations, namely, a smaller one centered at 15 nm and a second, larger and broader population centered at 50 nm. These two size distributions result in highly distinct nanoparticle volume distributions. Specifically, the distribution centered at 50 nm yields average volumes that are at least one order of magnitude larger than those of the distribution centered at 15 nm. In estimates of dye numbers, as well as in emission measurements (which are proportional to the volume), the contribution of the 15 nm-centered distribution is thus entirely negligible. Indeed, as the volume of the nanoparticles increases, the average number of dye molecules per nanoparticle increases as well, leading to a higher nanoparticle brightness. It will be later confirmed by the measured nanoparticle brightness distributions displayed in Figure 2b, where no significant peak can be observed for dFON(1) at very low intensities. We conclude that the emission from this population is too weak compared to the second population to have been measured. Therefore, all subsequent results can be interpreted as originating from the 50 nm-centered distribution.

The dyes constituting both nanoparticles share a similar structure, built around one benzothiadiazole acceptor moiety at their core and two diphenylamine donor moieties at their extremities. They only differ in the nature of the π bridge that connects the acceptor and donor moieties: In dye 1, the benzothiadiazole acceptor is flanked by two fluorene motifs bearing two *n*-butyl side chains each, whereas in dye 2, this bridge is extended by the addition of a thienyl motif between the benzothiadiazole and the fluorene, thus considerably redshifting its absorption and emission compared to dye 1.

### Optical properties of the nanoparticles

Despite the seemingly minor differences between dye 1 and 2, they differ significantly in their optical properties in solution, and in those of their associated nanoparticles, as illustrated in Figure 1 and Table 1. dFONs(1) were prepared from dye 1 and emit around 595 nm with a fluorescence quantum yield of 0.33, while dFONs(2), prepared from dye 2, emit around 689 nm with a lower quantum yield of 0.06, in agreement with the energy gap law [33]. It is worth noting that the fluorescence quantum yield of dFONs(1) has been reported in the literature to be independent of nanoparticle size [20]. This suggests that the excited states of the dFONs based on this quadrupolar structure are less affected by the aqueous environment. Furthermore, the 2P absorption cross sections of dFONs(1) and dFONs(2) differ vastly around λ_0_ = 1000 nm, with σ_2_(λ_0_)(1) ≈ 60 GM per dye molecule in dFONs(1) and σ_2_(λ_0_)(2) ≈ 930 GM per dye molecule in dFONs(2) [11]. For dFONs(2), the appearance of this second absorption band can be explained by dye 2’s different geometry allowing for non-centrosymmetrical conformations, leading to a more complex absorption profile than that of an ideal linear quadrupole. Importantly, the difference in size between dFONs(1) and dFONs(2) leads to a difference in the average number of dye molecules per particle, with 7500 dye molecules per dFON(1) and 2100 dye molecules per dFON(2). This results in dFONs(1) and dFONs(2) having similar 2P-excited brightness at λ_0_, *B*_2P_(λ_0_), respectively, 1.5 × 10^5^ and 1.2 × 10^5^ GM per nanoparticle, allowing for a direct comparison of their photostability under similar excitation conditions.

**Table 1:** Physical and photophysical properties of dFONs(1) and dFONs(2). λ_0_ = 1000 nm.

	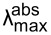 1P/2P^a^ [nm]	σ_2_(λ_0_)^b^ [×GM]	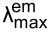 ^c^ [nm]	Φ^d^	Ø^e^ [nm]	N^f^	*B*_2P_(λ_0_)^g^ [×10^5^ GM]

dFONs(1)	462^h^/840^i^	60^i^	595^h^	0.33 ± 0.05^h^	29^h^	7500	1.5
dFONs(2)	532^i^/890^i^	930^i^	689^i^	0.06 ± 0.03^i^	20^i^	2100	1.2

^a^Absorption maxima wavelengths under one-photon (1P) and two-photon (2P) excitations ; ^b^two-photon absorption cross section of one molecule within dFONs at λ_0_ = 1000 nm; ^c^emission maximum wavelength; ^d^fluorescence quantum yield; ^e^mean dry diameter determined by transmission electron microscopy; ^f^theoretical average number of dye molecules per dFON (estimated from TEM measurements, assuming a density relative to water of 1.0); ^g^estimated mean two-photon brightness per dFON (calculated as the product of σ_2_(λ_0_), Φ and *N*); ^h^data from previous work [20]; ^i^data from previous work [11].

### Brightness of nanoparticles

The nanoparticles have been designed to emit the same signal intensity. This can be statistically demonstrated on single nanoparticles dispersed on a coverslip (see Experimental section). To do so, each coverslip was scanned using the piezoelectric stage with 100 nm steps and a 10 ms integration time per pixel. Laser excitation powers were maintained at 1.7 mW and 1.6 mW for dFONs(1) and dFONs(2), respectively. A typical scan is shown in Figure 2a, where several point spread functions (PSFs) are displayed corresponding to individual nanoparticles. We clearly observed different signal levels that can be related to size dispersion of individual nanoparticles and the presence of some aggregates (arrows on Figure 2a). The maximum detected photon counts for each bright spot, corresponding to a 2P-excited nanoemitter, were normalized by the square of the excitation power for direct comparison.

Histograms of emission signals from 293 individual dFONs(1) and 193 dFONs(2) (Figure 2b) revealed statistically similar distributions. Their comparison (see boxplots in Figure 2b) indicated comparable median positions (214 and 196 kcounts·s^−1^·mW^−2^, respectively) and quartile ranges. Mean values were very high (427 and 436 kcounts·s^−1^·mW^−2^, respectively), which is attributed to the potential aggregation of nanoparticles, leading to a few very high signals. A *t*-test yielded a *p*-value of 0.90, indicating no significant difference between the distributions. This consistent brightness across both dFONs validates the bottom-up synthesis design.

### Observation of the saturation and two-photon absorption cross section

**Figure 3:**
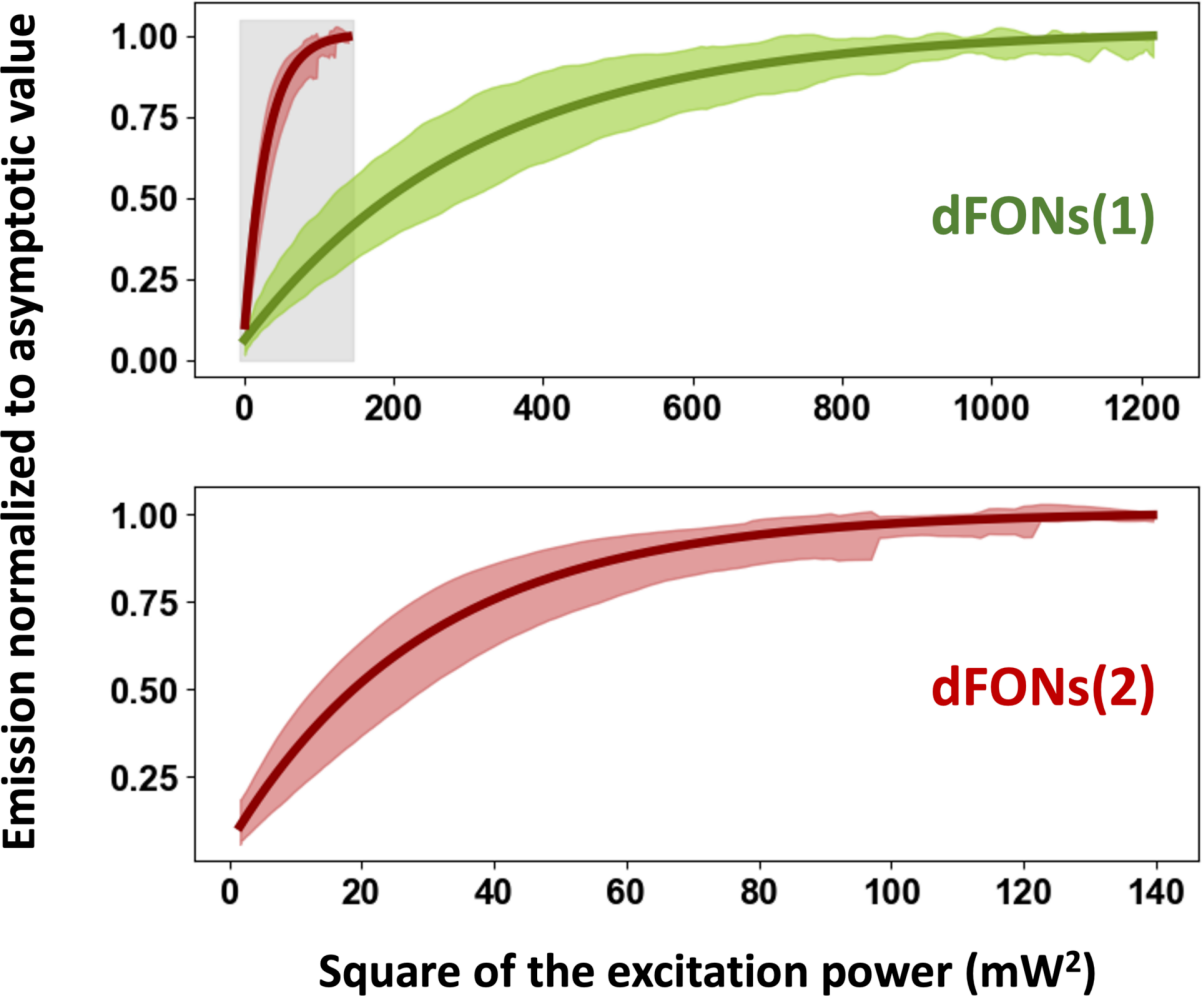
Saturation of the emission at high excitation powers for each kind of nanoparticle, dFONs(1) (top) and dFONs(2) (bottom). For each observed nanoparticle, we measured the emission as a function of the excitation power (e.g., half-wave plate position) and then calculated the emission normalized to its maximum value. Over a collection of nanoparticles, it led to a distribution of curves: Here, the colored, shadowed areas display this distribution (average signal ± one standard deviation). The plain line in each graph corresponds to the fit of the average value, following Equation 1. The saturation occurs at lower excitation power for dFONs(2) nanoparticles, whose results are reproduced in the grey area in the dFONs(1) curve.

Following the identification of individual nanoparticles, the laser focus was then maintained on each nanoparticle while the excitation power was modulated using a motorized mounted half-wave plate before a polarizer placed in front of the microscope. Fluorescence signals were recorded as a function of the half-wave plate angular position, which was subsequently converted into excitation power. Analysis across multiple nanoparticles provided measurements of the average signal and a standard deviation value for each excitation power. The curves, shown in Figure 3, exhibit clearly an asymptotic behavior, indicating saturation for high excitation power. For the dFONs(2) family, the saturation occurs at lower excitation powers than for dFONs(1). The fluorescence signals *F*(*P*_exc_), normalized to their asymptotic values, are fitted using Equation 1 (see Experimental section):


[1] $$F\left(P_{\text{exc}}\right)=1-\exp\left(-\frac{P_{\text{exc}}^{2}}{P_{\text{sat}}^{2}}\right),$$

where *P*_sat_ is the saturation power, which depends in particular on the nanoparticle capability to absorb the excitation light, and is thus related to the 2P absorption cross section of the individual dye molecules σ_2_.

Saturation powers were determined from the fits as 17.3 and 5.29 mW for dFONs(1) and dFONs(2), respectively. *P*_sat_ can be related to the 2P absorption cross section of the individual dye molecules, the incident beam characteristics (energy ℏω of a single infrared photon, waist *W*_0_ of the infrared excitation laser in the focal plane, repetition period *T* of the laser), and the characteristics of the dye assemblies (the 2P absorption cross section of individual dye molecules and, importantly, a coefficient α taking into account a local-field correction, that is, the electric field experienced by a single molecule into the nanoparticle). This local field is not only different from the incident field, but also from the one experienced by the NP under other conditions (when in water or in air, as in the current experiment). The following expression can be derived for each nanoparticle type using the developed model (see Experimental section for the derivation and estimated values of the parameters):


[2]

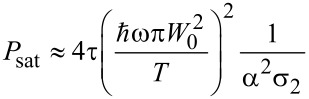



From the values of *P*_sat_, we find values of α^2^σ_2_ of 710 and 7580 GM for dFONs(1) and dFONs(2), respectively. These values have to be compared to those reported for the individual dye molecules within the nanoparticles (60 and 930 GM, respectively) [11]. The comparison leads to α ≈ 3.4 and α ≈ 2.9, which are values compatible with the expected refractive index from the dye aggregates [34–35] (see Experimental section).

### Bleaching of nanoparticles

Dye 2 demonstrated an approximately 14 times higher 2P absorption cross section compared to dye 1. The higher absorption probability in dFONs(2) implies that photobleaching should occur faster than in dFONs(1). Individual nanoparticle bleaching kinetics were thus monitored by directly recording fluorescence signals over time, fitted with a monoexponential decay to extract characteristic bleaching times τ*_b_*.

**Figure 4:**
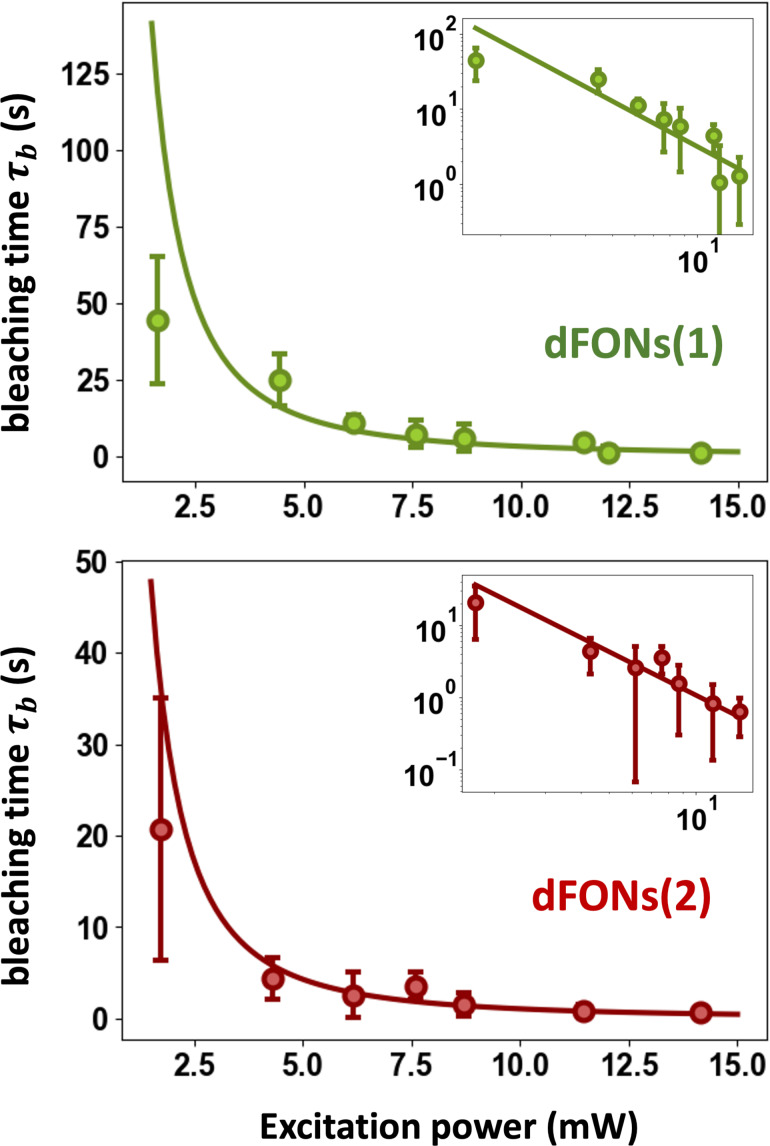
Characteristic bleaching time as a function of excitation power. At a given excitation power, the characteristic bleaching time from a monoexponential decay is calculated from the measurement over several nanoparticles. The results are reported with the average measured value (dots) and the standard deviation of the measurement for a given excitation (bars). The continuous line is a fit following Equation 3. Insets display the same graph in log-log scale: The linear behavior (slope −2) shows that the photobleaching time follows a 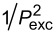 power law.

This experiment was conducted at various excitation powers on individual nanoparticles, yielding averaged results and standard deviations presented in Figure 4. Characteristic bleaching times τ*_b_* for dFONs(1) and dFONs(2) depended on 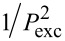 [21]:


[3] $$\tau_{b}=\tau_{b,0}{\left(\frac{P_{\text{sat}}}{P_{\text{exc}}}\right)}^{2}$$

The fit of the data determined τ*_b_*_,0_ values of 1.06 and 3.84 s for dFONs(1) and dFONs(2), respectively. However, this constant must be considered only with the value of *P*_sat_: At a given excitation power, the ratio of characteristic photobleaching times τ*_b_* corresponds indeed to the ratio of the quantity τ*_b_*_,0_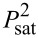. Hence dFONs(1) exhibited photobleaching times approximately three times longer than dFONs(2) under equivalent excitation conditions.

It is worth noting that smaller nanoparticles are commonly assumed to be inherently less photostable, albeit due to their higher surface-to-volume ratios and increased exposure of dye molecules to environmental interactions. We previously demonstrated a counterintuitive result: 14 nm NIR-emitting dFONs (fluorescence quantum yield Φ ≈ 0.01) were ten times more photostable in SPT than larger 43 nm green-emitting dFONs (Φ ≈ 0.14) [19]. This highlights that smaller size does not inherently correlate with lower photostability.

Among the various processes driving photobleaching, the formation of long-lived triplet states is recognized as a key factor in the rapid photobleaching of fluorescent dyes. To elucidate the role of triplet states in the photostability of dFONs(1) and dFONs(2), we measured the quantum yield of triplet state formation using singlet oxygen generation as a probe in molecular solutions of dyes 1 and 2 in toluene. Surprisingly, dye 1 was found to be a poor sensitizer, with a triplet formation quantum yield (Φ^Δ^) of 0.03. For dye 2, we were unable to detect any triplet state formation. This lack of reactive triplet states led us to conclude that the primary deactivation pathway for the S_1_→S_0_ transition in both dFONs is internal conversion, likely facilitated by the long alkyl chains and defects in the dye packing density within the nanoparticles. Such non-radiative deactivation is expected to significantly increase local heating.

Under 2P excitation, we hypothesize that the larger σ_2_ of dFONs(2) results in nanoparticle overheating due to rapid multicycling of excitation (induced by intense laser illumination and high repetition rates). This leads to thermal degradation of the dyes. Despite the smaller size of dFONs(2), their heat dissipation capacity may be insufficient to compensate for this intense excitation cycling. Consequently, we propose that two-photon-induced thermal effects represent the dominant mechanism responsible for the accelerated photobleaching of dFONs(2) [36].

## Conclusion

The comprehensive optical characterization of individual dFONs(1) and dFONs(2) confirms that both systems exhibit the expected comparable brightness under 2P excitation at λ = 1030 nm, despite differences in their molecular composition. Saturation measurements allowed to estimate the 2P absorption cross section of individual dye molecules aggregated in nanoparticles, in remarkable agreement with the values obtained with another independent method. The large value obtained for dFONs(2) correlates with a faster photobleaching rate, as confirmed by direct time-resolved measurements and their quadratic dependence on excitation power. Together, these results validate the bottom-up design strategy used for nanoparticle synthesis and highlight the trade-off between absorption efficiency and photostability. Such insights are essential for optimizing fluorophore-based nanoparticles in nonlinear microscopy and other bioimaging applications where brightness and stability must be carefully balanced.

## Experimental

### Nanoparticle preparation

The synthesis and characterization of the dyes are described in details in the Supplementary Information of [11]. 200 μL of dye solution of either 2 mM of dye 1 or 1 mM of dye 2 in THF were rapidly added to 19.8 mL of freshly distilled water at room temperature, under continuous sonication at 10 W – using a 20 kHz, 130 W ultrasonic processor (Vibra-Cell™ VC130, Sonics Instruments, Newtown, CT, USA), with a 6 mm diameter probe) – which was continued for 3 min after the addition, yielding, respectively, dFONs(1) or dFONs(2).

### TEM observation

The dry size of the dFONs was measured using TEM at the Bordeaux Imaging Center (BIC) facility. TEM images were acquired on a Hitachi H7650 at 80 kV. dFONs were deposited on copper grids previously charged (positively) by glow discharge technique. A drop of the dFONs colloidal solution was left on the charged grid for 1 min, and the excess of liquid was gently removed with a paper by capillarity. Then, a drop of aqueous uranyl acetate solution was added on the dFONs-loaded grid for 3 min. Afterward, the excess of liquid was again removed by capillarity. The size distribution was determined using ImageJ software.

### Coverslip preparation

A 10 µL droplet of individual nanoparticles was deposited onto a glass coverslip, which was then heated at 40 °C on a hotplate for 15 min to evaporate the solvent. 2P fluorescence signals from individual nanoparticles were observed using a custom-made inverted 2P microscope setup [22] with a water-immersion objective (NA 1.2, ×60, Nikon) and a piezoelectric nanopositioning stage (Piezoconcept) for sample scanning.

### Excitation and emission

Excitation was achieved using a femtosecond laser (τ = 135 fs typical pulse duration, wavelength of 1030 nm, 80 MHz repetition rate, Spark laser). The laser was focused near the diffraction limit onto the sample, leading to a point spread function (PSF) that could be fitted by a symmetric 2D Gaussian function with a standard deviation of about 250 nm, equivalent to a waist *W*_0_ ≈ 360 nm for the infrared beam. Fluorescence was collected by the same objective, separated from the excitation using a dichroic filter (FF749, Semrock) before being sent to a photon counting module (SPCM AQRH-13, Picoquant). Two additional filters (FF01-842/SP25 and FF01-945/SP25, Semrock) were added to remove residual laser light. The combination of all the filters leads to the collection of approximately 80% of the fluorescence for both nanoparticle families.

### Emission model (saturation and 2P absorption cross section)

Emission behavior can be calculated using rate equations for populations of the excited and ground states. For the sake of simplicity, a pulse of the excitation laser is modeled with a rectangular shape of duration δ*t* at a periodicity *T*. Taking τ the typical duration of the sech^2^ function describing the real pulse, the rectangular model has a δ*t* = 2τ to get the same integral value over time. The excitation rate Γ_exc_ during the pulse can be expressed as a function of the average power *P*_exc_, the two-photon absorption cross section σ_2_, and the waist of the excitation laser at the nanoparticle position *W*_0_:


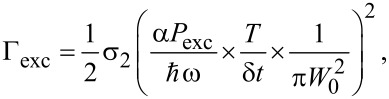


where the factor 1/2 is related to the absorption of two infrared photons for each excitation event, *P*_exc_*T*/δ*t* is the incident peak power, and α is a coefficient taking into account a local-field correction, that is the electric field **E**_loc_ experienced by a single molecule into the nanoparticle. The order of magnitude of such local-field correction can be given by the so-called Lorentz model:


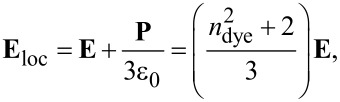


where **P** = ε_0_(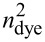 − 1)**E** is the polarization vector. It leads to a coefficient α = ((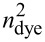 + 2)/3)^2^. Considering a refractive index of the pure dye ranging from 1.5 to 1.8 [34], and even up to 3 near an absorption resonance [35], α is between 2 and 13. We point out the fact that local fields exist also in the measurements of σ_2_ and Φ from Table 1. The corrections change, however, depending on the dFONs environment. As a result, the values of α measured in the paper cannot be explained only by a single Lorentz factor, but they fall into the expected order of magnitude.

With Γ the radiative decay rate, integrating the rate equations over *T* lead to a fluorescence signal varying as:


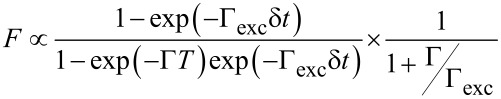


In our experiment Γ_exc_ ≫ Γ, so that the emission can be just written as:


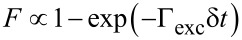


It is straightforward to obtain the expression of *F* versus *P*_exc_ (Equation 1 of the main text) introducing a saturation term *P*_sat_ such as:


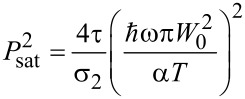


## Data Availability

Data generated and analyzed during this study is openly available on Zenodo, reference number 17610635, at https://doi.org/10.5281/zenodo.17610635.
